# Supplementary material for: Optimal experimental design for partially observable pure birth processes
Source: PLoS One. 2025 Aug 29;20(8):e0328707. doi: 10.1371/journal.pone.0328707 (PMC12396769; doi:10.1371/journal.pone.0328707)
Supplement: Attachment — Submitted filename: Reports on PONE-D-24-59633.pdf [file pone.0328707.s001.pdf]

Reports on "PONE-D-24-59633"

Title: Optimal experimental design for partially observable pure birth processes
Authors: By Ali Eshragh, Matthew Paul Skerritt, Bruno Salvy and Thomas McCallum

This work addresses the optimal experimental design for partially observable pure birth processes (POPBPs), aiming to maximize the Fisher information with respect to the birth rate parameter. The authors introduce an innovative computational approach that leverages generating functions and symbolic computation to efficiently compute the Fisher information for these complex stochastic processes. This study is clearly relevant to researchers in stochastic modeling, applied probability, and statistical inference.

The application of generating function techniques to optimize Fisher information in partially observable birth processes is novel and represents a significant advancement over previous methods that were restricted to small values of n. The recursive formulation provides a practical computational tool, enabling computations for larger values of n that were previously intractable. The theoretical derivations are well-structured, with clear formulations of generating functions, recurrence relations, and their implications for the likelihood and Fisher information. The provision of a publicly available C++ implementation and GitHub repository further enhances the reproducibility and potential impact of the work.

In conclusion, this work is novel, interesting, and meaningful. Moreover, the presentation of this paper is clear and the proofs and computations appear to be correct. Therefore, I recommend that the paper be accepted for publication in *PLOS ONE*.

However, the authors should carefully proofread the manuscript, as there are still some typographical and grammatical errors. For example:

1. The notation involving multiple indices (e.g., $\overline{y}_n$, $\overline{c}$, $q_{\overline{c}}$) may be overwhelming for readers unfamiliar with combinatorial generating functions. Consider adding a summary table of notation for reference;

2. Figure references (e.g., Fig. 1, Fig. 11) should be cross-checked to ensure correct linkage and appropriate caption placement;

3. Page 21, line 711: "of of six days" $\longrightarrow$ "of six days";

   ...
