# Supplementary material for: Optimal experimental design for partially observable pure birth processes
Source: PLoS One. 2025 Aug 29;20(8):e0328707. doi: 10.1371/journal.pone.0328707 (PMC12396769; doi:10.1371/journal.pone.0328707)
Supplement: Attachment — Submitted filename: Response to Reviewers.pdf [file pone.0328707.s002.pdf]

# Comments in Response to the Referees' Reports on "Optimal experimental design for partially observable pure birth processes"

Ali Eshragh, Matthew P. Skerritt, Bruno Salvy, Thomas McCallum

We sincerely thank the Editor and the two reviewers for their thoughtful and constructive feedback. We are grateful for their positive assessment of our work. We have carefully addressed all the comments raised, and we believe the revisions have improved the clarity and presentation of the manuscript. For ease of reference, the changes made in response to the comments have been highlighted in blue in the revised version. Below, we provide a detailed response outlining how each of the points in the referee report has been addressed.

## Responses to Editor

We thank you so much for considering our work and taking the time to read it carefully. We have outlined below how we have fully addressed all the comments.

## Responses to Reviewer #1

We thank you very much for your positive evaluation of our work and your recommendation for acceptance. We really appreciate your encouraging feedback.

## Responses to Reviewer #2

We thank you very much for your positive evaluation of our work and your recommendation for minor revision. Your encouraging feedback and constructive suggestions were greatly appreciated and have been instrumental in helping us enhance the clarity and quality of the manuscript. Below, we provide a detailed explanation of how each of your comments has been addressed.

1. *The authors should carefully proofread the manuscript, as there are still some typographical and grammatical errors.*

We have carefully proofread the entire manuscript and identified a few minor typographical errors, which are listed below. These, along with the items kindly noted by you, have all been corrected and are highlighted in the revised version.

(i) "optimisation" → "optimization" in the caption of Figure 2.

(ii) "grey" → "gray" on lines 565 and 566 of page 16.

2. *The notation involving multiple indices (e.g., $\overline{y}_n$, $\bar{c}$, $q_{\bar{c}}$ ) may be overwhelming for readers unfamiliar with combinatorial generating functions. Consider adding a summary table of notation for reference.*

   We have added a new Table 1 at the end of the Introduction section to introduce all notation used throughout the paper. We hope this improves clarity and readability for the reader.

3. *Figure references (e.g., Fig. 1, Fig. 11) should be cross-checked to ensure correct linkage and appropriate caption placement.*

   We have carefully reviewed all figure references and captions throughout the manuscript to ensure consistency and correct placement. All figures are appropriately referenced, and their captions match the correct content. While we have ensured that captions appear immediately after the first mention of each figure, LaTeX may occasionally place a figure or its caption at the top of the following page (e.g., Figure 11). We have done our best to minimize such instances while adhering to journal formatting conventions.

4. *Page 21, line 711: "of of six days" ⟶ "of six days".*

   We have removed the redundant "of".
